# Improving Protein Quantity and Quality—The Next Level of Plant Molecular Farming

**DOI:** 10.3390/ijms23031326

**Published:** 2022-01-25

**Authors:** Hai Liu, Michael P. Timko

**Affiliations:** Department of Biology, University of Virginia, Charlottesville, VA 22904, USA; hl9h@virginia.edu

**Keywords:** plant molecular farming, genetic engineering, recombinant protein expression, secretion pathway, deconstructed vectors, virus-like particles, *N*-glycosylation, *O*-glycosylation

## Abstract

Plants offer several unique advantages in the production of recombinant pharmaceuticals for humans and animals. Although numerous recombinant proteins have been expressed in plants, only a small fraction have been successfully put into use. The hugely distinct expression systems between plant and animal cells frequently cause insufficient yield of the recombinant proteins with poor or undesired activity. To overcome the issues that greatly constrain the development of plant-produced pharmaceuticals, great efforts have been made to improve expression systems and develop alternative strategies to increase both the quantity and quality of the recombinant proteins. Recent technological revolutions, such as targeted genome editing, deconstructed vectors, virus-like particles, and humanized glycosylation, have led to great advances in plant molecular farming to meet the industrial manufacturing and clinical application standards. In this review, we discuss the technological advances made in various plant expression platforms, with special focus on the upstream designs and milestone achievements in improving the yield and glycosylation of the plant-produced pharmaceutical proteins.

## 1. Introduction

Using plant-based platforms for the production of high-value recombinant proteins, also referred to as plant molecular farming, has been a promising concept since the 1980s, with the goal of making plant-produced pharmaceuticals an alternative for industrial and clinical applications [1,2]. Despite several proposed advantages of plants over other biologically-based production systems [3,4,5], the early stages of plant molecular farming advanced slowly and its products had small competitive advantages in human clinical trials due to technological restrictions and limited knowledge [6]. During the past two decades, great progress has been made towards the development of plant-made pharmaceuticals, such as antibodies, vaccine antigens, hormones, and antimicrobial peptides (AMPs) [4,5,7,8,9]. The development of plant expression constructs (e.g., deconstructed viral replicon-based vectors) has substantially enhanced the recombinant protein yield in plant cells and made rapid and largescale production possible [10,11]. Plant-produced virus-like particles (VLPs) exhibit great potential of presenting the recombinant proteins on the surface of these self-assembled nanoparticles, which have opened a new realm for synthetic vaccine production and biomedical applications [12,13,14,15,16]. Glycosylation is one of the most important post-translational modifications (PTMs) crucial to the clinical values of many pharmaceutical proteins, such as antibodies and vaccine antigens. Considerable effort has been made to eliminate plant specific glycan structures and introduce human-type glycosylation pathways into plants, and these glycoengineered plants are now able to generate structurally authentic glycoproteins [17,18]. These revolutionary advances in science and technology have led to big breakthroughs in plant molecular farming, with more and more products advancing to late-stage clinical trials and beyond [3,19]. There are also many plant-based products, such as veterinary pharmaceuticals, technical and research reagents, and cosmetic ingredients, that are already commercially available [19].

Like many developed commercial production platforms that utilize different expression hosts (e.g., mammalian cells, *Escherichia coli*, and yeast) with respective advantages and disadvantages, plants also offer diversified expression platforms to meet various requirements, such as lead time, yield, glycosylation, storability, or scalability, of the recombinant proteins [20]. Utilizing plant cell cultures (e.g., tobacco BY-2) or tissue cultures (e.g., tobacco hairy roots) as the production platforms for recombinant proteins has many advantages over whole plant transformation systems, and even the mammalian and bacterial systems, including cost-effective cell culture, rapid scale-up, high biomass, and low risk of animal or human pathogenic contaminations [3,21,22]. Recombinant human β-glucocerebrosidase (taliglucerase alfa) produced in carrot suspension cells culture was the first FDA-approved plant-made therapeutic protein to treat human disease [23,24]. Recently developed cell packs derived from tobacco BY-2, a form of medium-deprived porous plant cell aggregates, showed very promising efficiency and scalability for recombinant protein production and, when used together with the plant viral replicon-based constructs, the platform could achieve much higher yields of transiently expressed proteins [25,26]. Transient expression in fresh plant leaf tissue is another frequently chosen strategy that allows the fast and robust production of recombinant proteins in a matter of weeks or even days. *Nicotiana benthamiana* is one of the most widely used plant materials for the transient expression of recombinant proteins due to its many favorable attributes, such as fast growth, large biomass, robust reproduction, and easy scalability. It is a versatile platform for either quick assays on proof-of-concept designs, or variable scales of fast production of final products, especially in response to epidemic and pandemic threats. On the one hand, the stable transformation (both genetic and transplastomic) of plants is often used to introduce additional agronomic attributes for crop protection or nutritional improvement. On the other hand, it provides a more sustainable and scalable solution for the mass production of recombinant pharmaceuticals [1,27,28]. Therapeutic proteins, such as antigens and antibodies, can be expressed and accumulated in the edible parts of the plant, such as the seeds or grains of cereals and legumes, tap roots and tubers, fruits, and leaves of fresh produce, for easy oral delivery, which not only provides the benefits of easy administration to both human and veterinary pharmaceuticals, but also greatly reduces the production cost by minimizing downstream purification and modification [19,28,29]. In addition, seeds naturally provide a very stable environment for protein storage even under ambient conditions, which means that the seed-based pharmaceutical products can even be stored and distributed in a cold-chain-free manner [2,19,30].

In this review, we highlight the technological advances made in the improved yield and increased quality of plant-made pharmaceutical proteins and discuss how these breakthroughs led to greater industrial and clinical applications from plant molecular farming.

## 2. Strategies to Improve Protein Yield

Despite the similarities shared between plants and mammals in their protein secretory pathways (e.g., protein folding, trafficking and glycosylation), low yields and/or diminished biological activity of many recombinant proteins produced in plant cells are still the biggest challenges faced in cross-kingdom expression due to incompatibilities arising from factors such as codon bias [31,32], incorrect or insufficient folding [33,34], non-humanized *N*-glycosylation [7,17], host silencing effect [35,36], and proteolytic degradation [37,38]. To address these issues, great progress has been made by focusing on various aspects of the protein production process.

### 2.1. Codon Optimization

It is now clear that codon biases can have a profound impact on the protein expression in heterologous host organisms [39,40,41]. Generally, most of the pharmaceutically relevant recombinant proteins expressed in plants are of human genome origin or from human pathogens. Therefore, the coding sequences of these proteins are likely to contain codons whose use frequencies match human cellular activities rather than those in plants. The consequence of such mismatches in codon use is translational pause or abortion. Codon optimization of recombinant protein sequences in favor of the codon usage of the expression hosts has been shown in numerous studies as an effective means of increasing translation efficiency and protein yield. In a recent study, codon optimization led to a 25- to 30-fold increase in the yield of a recombinant stem cell factor (SCF) protein in tobacco BY-2 cells [42]. In contrast, there are also contradictory reports of the effects of codon optimization. For example, codon optimization of human erythropoietin (EPO) or interferon gamma (IFNγ) genes for expression in plants exhibited little advantage in terms of protein yield [43,44]. Similarly, studies on the optimization of codon usage for the expression of HPV-16 L1 capsid protein in *N. benthamiana* found that the native genes with human codon usage worked better than those optimized to reflect plant codon usage [45,46]. Recently, it has been shown that in some cases codon optimization may affect mRNA structural profiles that are necessary for effective protein synthesis. Understanding codon usage and the size of tRNA pools in both the source and host organisms can also assist in creating harmonized codons for enhanced translational efficiency [47].

### 2.2. Promoters, Terminators, and Untranslated Regions (UTRs)

Proper gene transcription initiation, termination, and message polyadenylation are key components of gene expression and having the right combination of gene promoters and terminators is critical in constructing proper transgene expression cassettes. The effective polyadenylation by terminators has a great impact on gene transcription and mRNA processing for translation from nucleus to cytoplasm [48]. Both 5′- and 3′-UTRs also play an important role in determining mRNA stability and translation [49,50,51]. Both constitutive and cell/tissue-specific type promoters have been routinely used in various plant heterologous expression systems. Strong and robust constitutive promoters are usually preferred in the transient expression systems for the maximum transcript accumulation and protein yield. These include the cauliflower mosaic virus (CaMV) 35S promoter, the most commonly used promoter, plant-derived ubiquitin promoters, and the Agrobacterium-derived nopaline synthase (NOS) promoter [52]. The CaMV35S promoter and NOS terminators are among the most universally adopted pairing of regulatory sequences found in plant expression constructs. A number of newly identified terminators and terminator-containing 3′-UTRs, such as the Arabidopsis heat shock protein (HSP) terminator, the soybean vspB terminator, the tobacco intronless extensin terminator (EU), and the *N. benthamiana* Actin3 (NbACT3) terminator, have been demonstrated to be much more efficient than the NOS terminator in target protein expression [48,50,53,54]. Through comparative analysis of various promising 5′-UTRs and 3′-UTRs (including terminators), several were demonstrated to be highly effective in the enhanced recombinant protein production in plants. For example, the use of Arabidopsis alcohol dehydrogenase (ADH) 5′-UTR together with the Arabidopsis heat shock protein (HSP) terminator gave the best yield and highest activity of the recombinant human β-glucocerebrosidase among the tested expression constructs in *N. benthamiana* [55]. Two truncated 5′-UTRs from the Arabidopsis and *N. benthamiana* photosystem I K (PsaK) genes exhibited a better capability of enhancing protein production than the famous tobacco mosaic virus (TMV) omega leader sequence, while EU provided more than 10-fold increase in protein yield compared to the NOS terminator [48,50]. In addition, many terminators exhibited synergistic effect when two were combined in tandem, leading to more than doubled protein yield compared to being individually used, such as CaMV35S terminator combined with NOS terminator, EU combined with NbACT3 terminator, and HSP terminator combined with tobacco extension terminator [48,56,57]. Interestingly, an attempted triple terminator concatenation showed decreased protein expression when compared to the double terminator setups [57], suggesting largely unknown molecular mechanisms of these 3′-UTRs.

For the production of pharmaceutically-relevant proteins in crops, especially cereals, tissue-specific promoters were preferred more than the constitutive ones for targeted high-level and stable protein yield while not affecting the normal growth and development [46]. Several recombinant proteins, such as antigens, antibodies, and AMPs, have been efficiently expressed and accumulated in the seeds of stably transformed cereal plants, such as rice, corn, and barley (Table 1). The gene promoters of the native seed storage proteins, including glutelin (GluB-1 and GluB-4), prolamin, and globulin (Glb-1), are strong and specific promoters usually used for protein expression in the endosperm tissue of rice seeds [40]. Several endosperm-specific promoters have also been successfully used across species. In maize, the rice GluB-1 promoter combined with the intron1 of the maize ubiquitin-1 promoter led to the successful expression of the recombinant anti-HIV-1 monoclonal antibody (mAb) 2G12 in the endosperm cells [58]. A much higher yield of 2G12 was reported in the endosperm of transgenic barley using the oat GLOBULIN1 promoter [59]. Recently, biologically active human AMP LL-37 was produced in stable transgenic barley. The native seed-specific promoter of barley B1 hordein gene showed great superiority over the maize ubiquitin promoter in terms of both the yield and endosperm-specific accumulation of LL-37 [60]. Due to the long lead times for the development of stable and homozygous transgenic lines, comprehensive comparisons between different promoters, 5′-UTRs, and terminators on a large scale may seem unrealistic. Therefore, correctly choosing promoters for specific tissue accumulation will be pivotal to the effective production of recombinant proteins in stably transformed plants.

### 2.3. Subcellular and Apoplast Targeting

Previous studies have reported that the subcellular and apoplast localization of a recombinant protein can have a dramatic impact on its accumulation level. Compared to cytosol localization, recombinant pharmaceutical proteins, such as vaccine candidates, antimicrobial peptides, and antibody fragments, generally have a much higher yield when targeted to the apoplast, the endoplasmic reticulum (ER), or the chloroplast, most likely due to the less proteolytic activities at these locations, in addition to the facilitated folding and glycosylation along the secretory pathway [7,37,78,79,80,81].

#### 2.3.1. Apoplast

A secretion signal peptide (SP), the prerequisite for protein secretion, is a short (15–30 aa) peptide most often located at the N-terminus that directs entrance of the protein into the endoplasmic reticulum (ER) lumen, where the protein is folded and packed for the transportation to the Golgi apparatus, the organelle for further modifications (e.g., *N*-glycosylation) and the eventual release of the protein to the apoplast. Targeting the recombinant protein to the apoplast is extremely popular across plant cell culture platforms, such as the tobacco BY-2 cell culture, tobacco hairy root culture, and rice cell culture, because it can lead to a relatively high yield of the target protein directly secreted into the culture media, facilitating downstream purification processes [82]. Most of the recombinant proteins (e.g., human growth hormone, interferon α2, recombinant antigens and antibodies) that were recently made in tobacco BY-2 cell culture were engineered to be secreted into the culture medium [21]. Table 2 lists examples of plant-derived SPs for the expression of recombinant biopharmaceutical proteins in plants. Notably, a recently identified rice SP, 33KDsp, was demonstrated to be more efficient than the commonly used αAmy3sp/Ramy3sp at promoting the secretion of recombinant proteins in rice suspension cells [83]. Similarly, through a parallel comparison with several commonly used SP sequences, the native extensin secretory signal NbSS^Ext^ was highlighted to be the most efficient one in terms of recombinant protein yield and secretion in *N. benthamiana* [84]. However, its closest counterpart, the NtSS^Ext^/SS^tob^ from *N. tabacum* (differing from NbSS^Ext^ by only one amino acid residue, Table 2), was not included in this comparison considering the successful stories of the NtSS^Ext^/SS^tob^ used for the expression of various recombinant pharmaceutical proteins [42,85,86,87]. Interestingly, some mammal native SPs can also be recognized and accurately processed in plant cells. For instance, the native SP of human growth hormone (hGH) led to the correct processing and apoplast targeting of active hGH in *N. benthamiana* [88], and the native human acid-alpha glucosidase (GAA) SP was shown to be fully capable of facilitating the entrance of GAA into the ER lumen, leading to the eventual secretion into the Arabidopsis cell culture medium [89]. The native murine mAb24 heavy chain-derived signal peptide (LPH) was also frequently used for the expression of viral glycoproteins [90].

Although not directly related to secretion signal peptide structure or composition, recent studies have found that the composition of the cell or tissue culture medium used can have a significant impact on recombinant protein yield. Customized optimizations of the culture media have resulted in boosted secretion and enhanced stability of recombinant proteins [91,92,93].

#### 2.3.2. ER

Many pharmaceutically important proteins, such as antigen or antibody fragments, are species-specifically glycosylated when produced naturally. Although ER-associated glycosylation generates high mannose-type *N*-glycans believed to be identical in plants and mammals, the complex *N*-glycan structures formed in the plant Golgi apparatus (e.g., β1,2-xylose and core α1,3-fucose residues) are distinct from those in mammals and are generally believed to be potential sources of allergenicity for human [108]. One circumventing strategy is to target the recombinant protein for retention in the ER by including both an N-terminal SP and a C-terminal ER retention signal HDEL/(SE)KDEL, thereby minimizing the formation of plant-specific *N*-glycans. Additionally, the ER mainly functions for protein folding and sorting, but not degradation *per se*, and is believed to host very limited types of proteases [109]. Although the presence of oligomannosidic *N*-glycan structures sometimes still raises concerns due to their possible negative effects on the efficacy of recombinant pharmaceuticals, the use of ER-retention is still widely used for both the transient expression and stable genetic transformation platforms because it is an easy fix to increase the yield and stability of many recombinant proteins [2,78].

For decades, many recombinant proteins with pharmaceutical potential have been produced and retained at ER through transient expression in *N. benthamiana* (Table 3). The increased accumulation and improved stability of ER-accumulated pharmaceutical proteins have also been reported in various stable transgenic plant species (e.g., Arabidopsis, tobacco, tomato, potato, and sugarcane) [110,111,112,113,114,115]. In cereal crops, there are two major organelles responsible for protein storage in the seed endosperm cells: (i) ER-derived protein bodies (PBs), mostly for prolamin accumulation, and (ii) post-Golgi protein storage vacuoles (PSVs) containing globulins and glutelins. Both are generally believed to possess relatively low water contents and low protease activity [2,40,58]. Thus, these highly specialized protein storage organelles in cereal endosperm tissue provide isolated microenvironments for the stable accumulation and long-term storage of recombinant proteins. To date, pharmaceutical proteins with acceptable yields and bioactivity have been produced in the seeds of stable transgenic cereal plants, mostly rice, barley, and corn (Table 1).

Nevertheless, erratic deposits of ER-targeted recombinant proteins have also been reported. In the seeds of transgenic Arabidopsis and tobacco, several ER-targeted recombinant antibodies have been found to be partially secreted or sorted to the PSVs, where they may subject to unwanted glycosylation or suffer specific proteolytic degradation [126,127,128]. Similarly, the ER-targeted recombinant protein CMG2-Fc was detected in the culture media when expressed in a glycoengineered *N. benthamiana* suspension cell culture [129].

#### 2.3.3. Vacuole

The most successful case of a vacuole-targeted recombinant protein may be recombinant human β-glucocerebrosidase (taliglucerase alfa) produced in carrot cell culture using the storage vacuole targeting signal DLLVDTM from tobacco chitinase A [130]. In 2012, the purified recombinant human β-glucocerebrosidase (marketed by Protalix Biotherapeutics under the name of Elelyso) became the first FDA-approved plant-made pharmaceutical [23] for enzyme replacement therapy of Gaucher disease. To be effective, β-glucocerebrosidase requires certain PTMs that can generate appropriate terminal mannose residues for the uptake by the target cells. The production of Elelyso takes full advantage of the plant vacuole *N*-glycosylation pattern that yields paucimannosidic-type *N*-glycan structures with exposed mannose residues. Clinical trial data indicate that the plant produced enzyme has efficacy equivalent to those produced in mammalian cells (e.g., Cerezyme (imiglucerase, by Genzyme Corporation) and VPRIV (velaglucerase alfa, by Shire Pharmaceuticals)) [131]. The advantages of Elelyso production in carrot cells, such as the naturally produced mannose-terminated glycan structure, low risk of mammalian pathogen transmission, and the cost efficiency and easy scalability, make it a very promising substitute for the treatment of Gaucher disease [24].

During an investigation into the effects of subcellular localization on recombinant protein expression, various versions of recombinant *Aspergillus niger* polygalacturonase I (AnPGI) were transiently expressed in *N. benthamiana* and evaluated for accumulation in different cell organelles and the apoplast. The enzyme activity of vacuole-targeted AnPGI was found to be the highest, although its accumulation in the vacuole was not as high as that found in the apoplast or ER. This finding suggested that a vacuole specific post-translational modification may have a positive effect on the activity of AnPGI [132]. Recently, a wheat-derived vacuole targeting sequence was identified and used to successfully target three stable proteins (i.e., GFP, GUS, and aprotinin) to the vacuoles of sugarcane stem parenchyma, leading to high yields of recombinant protein from the sugarcane juice [80]. Therefore, plant vacuole can also be a desired targeting candidate for recombinant protein expression, especially when a certain type of post-translational modification is required for the anticipated protein activity.

#### 2.3.4. Chloroplast

By the N-terminal fusion of a chloroplast transit peptide (CTP), such as that found on the RuBisCO small subunit (RbcS) precursor, recombinant proteins are typically targeted to the chloroplast for accumulation. This strategy often has a dramatic positive effect on protein accumulation and, as a consequence, has been widely used, especially for the development of plant-based vaccines for human and animals [46]. The versatility and cost efficiency of plant expression systems has made them an attractive option for the production of vaccines to high-risk viruses where there is a large worldwide demand for such products and high production cost for current commercial vaccines [4,20,133]. For example, for the development of plant-based vaccines against the human immunodeficiency virus type 1 (HIV-1), responsible for 0.5–1.0 million deaths annually (https://www.who.int/news-room/fact-sheets/detail/hiv-aids, accesseded on 30 November 2021), the recombinant HIV-1 capsid protein p24 was fused with the matrix subunits p17 (p17/p24) and expressed both transiently and stably in tobacco. In both cases, chloroplast targeting led to the highest protein yield of p17/p24 compared to being targeted elsewhere in the cell, and, more importantly, the plastid accumulated p17/p24 was able to elicit humoral and T cell immune response in mice [134]. Another example is the human papillomaviruses (HPVs) that contribute to more than 99% of cervical cancer cases, one of the most common of the cancers that are greatly threatening women health worldwide [135]. Maclean et al. (2007) first reported the high accumulation level of the HPV-16 L1 capsid protein in the chloroplast in both transient expression (up to 17% TSP) and stable transgenic (up to 11%) tobacco [45]. This finding was later corroborated by a similar, independent study in tobacco in which the chloroplast-targeted expression of HPV-16 L1 was found to have the greatest yield and formed VLP structures that resemble the commercial VLP-based HPV vaccines [136]. Subsequently, the recombinant HPV-16 E7 oncoprotein fused with a *Limulus polyphemus* anti-lipopolysaccharide factor fragment (LALF_32–51_) was produced in transiently expressed *N. benthamiana* leaves as the vaccine candidate with both protective and therapeutic qualities. The recombinant LALF_32–51_-E7 exhibited a dramatically higher yield (27-fold) when targeted to the chloroplast compared to the cytoplasmic-localized version [81,137].

Since the development of stable chloroplast transformation/engineering in the early 1990s, the great potential of the chloroplast as a factory for recombinant protein production has become realized [138]. Each plant mesophyll cell contains between 40 to more than 100 chloroplasts, with each chloroplast containing 15 or more copies of the chloroplast genome. Therefore, each leaf cell has copies of the chloroplast genome ranging in number from thousands to tens of thousands, which makes chloroplast transformation highly advantageous compared to traditional nuclear transformation [139,140,141]. In a stable transplastomic plant, the high copy number of the transgene in the transplastomic cells compared to that in the nucleus-transformed cells is believed to give exceptionally high expression of the recombinant protein. In addition, the expression cassette is usually integrated into a precise intergenic region (e.g., between the *rbcL* and *accD* genes) of the chloroplast genome through sequence specific homologous recombination, thus leaving the functional integrity of the chloroplast genome uninterrupted [138]. Indeed, a large number of nutraceuticals and pharmaceuticals, including antioxidants, AMPs, and vaccine antigens, have been successfully produced in several transplastomic crop species for the past two decades, and they generally showed higher yields than those produced by traditional genetic transformation [138]. Other unique features of the chloroplast transformation include: (i) it is free of epigenetic or silencing effects thereby allowing for consistent transgene expression; (ii) the bacterial-like polycistronic transcription units and translation machinery allow for multiple transgenes to be expressed in a single operon; and (iii) the maternal inheritance of the chloroplast genome prevents transgene escape via pollen, thus minimizing transgene contamination, one of the major concerns for the nuclear transgenic plants in agriculture [138,142,143].

Despite being a very promising platform for the large-scale production of various biopharmaceuticals, there are drawbacks associated with chloroplast transformation that currently limit its broad implementation. First, due to the high divergence of the chloroplast intergenic and regulatory sequences among plant species, chloroplast transformation vectors must be specifically constructed for each species [138]. Only plant species with well-characterized chloroplast genomes are considered as feasible candidates for chloroplast transformation and the whole process is subject to a long lead time [144]. Second, the bacterial-like translation machinery of the chloroplast utilizes a minimal set of tRNAs that may pose translational constraints for some foreign transgenes [142]. Finally, the chloroplast-expressed proteins are retained within the organelle, where there are very limited eukaryotic-style PTMs (e.g., glycosylation). Therefore, for the expression of recombinant proteins that require complex glycosylation for proper function or secretion, chloroplast transformation may not be the ideal strategy [144,145].

In summary, the best site (i.e., cytosol, chloroplast, ER, vacuole, and apoplast) for targeting recombinant proteins to achieve stable and robust accumulation can only be determined empirically because of the unique characteristics and peculiarities of each protein to be expressed. The microenvironments at different subcellular locations tend to have different PTMs, pHs, and proteolytic activities, which may favorably or adversely affect the stability and bioactivity of the recombinant protein. Therefore, various versions of the expression constructs for each protein of interest should be experimented with and evaluated in every aspect before a final verdict to be made for practice.

### 2.4. Fusion of Protein Partners/Carriers/Peptide Tags

The heterologous expression of the recombinant proteins in plant cells often leads to low to no soluble protein yield, which may be the result of insoluble aggregation or rapid premature degradation of the target protein due to the limitations of the PTMs within plant cells, such as underglycosylation and aberrant folding [18,109]. Largely, this resembles the situation in *E.* *coli*, which lacks PTMs and faces persistent issues with target protein expression and solubility. Nevertheless, it is still used extensively in processes ranging from laboratory-scale research to large-scale production of potential biopharmaceutical proteins [146]. For decades, scientists have made great progress using *E. coli*-based systems for the production of stable and active recombinant proteins. This includes the creation of a variety of peptide tags and protein partners or chaperons to be fused with the target proteins to improve their expression and solubility, and to aid in downstream purifications [147]. The glutathione-S-transferase (GST), maltose-binding protein (MBP), thioredoxin (TRX), and small ubiquitin-related modifier (SUMO) are well-known protein partners widely used for enhancing protein solubility and expression. In addition, GST and MBP also serve as purification tags to be used alone or in combination with other affinity tags, such as c-myc, Flag, 6xHis, HA, and StrepII [148,149]. In plants, there seem to be few reports on the effects of these solubility enhancing protein partners on promoting the expression of heterologous fusion proteins. A recent study, however, reported a negative effect of SUMO fusion on the transient expression of a human antimicrobial peptide LL-37 in tobacco, and that the fusion of MBP to LL-37 did not have a discernible positive influence on the protein yield [60]. Nevertheless, several promising protein fusion partners have been discovered to enhance the accumulation of recombinant proteins in plants.

Zera^®^, the N-terminal proline-rich region from maize γ-zein, which primarily contains eight repeats of the PPPVHL hexapeptide unit, has been shown to effectively stabilize several pharmaceutical proteins, such as calcitonin (Ct), epidermal growth factor (EGF), and human growth hormone (hGH), by the intracellular encapsulation into the ER-derived PB-like organelles in plant and other eukaryotic cells [150]. The recombinant protein vaccine made of the E7 protein of HPV fused with Zera^®^ was successfully produced in *N. benthamiana* with high yield and specific immunogenicity [151]. In addition, the elastin-like polypeptides (ELPs) are repeats of the pentapeptide sequence VPGXG, where X stands for any amino acid except proline and the number of repeats can range from 5 to 160 [152]. ELP fusion has been demonstrated to significantly enhance the accumulation of a number of heterologous recombinant proteins, such as mAbs, interleukins (ILs), EPO, and AMPs, in ER-derived BPs in both transiently and stably transformed tobacco [125,152,153,154,155]. The thermal responsive characteristics of ELP facilitates the easy purification of fusion proteins with a simple inverse transition cycling method [152,156]. HFBI, a hydrophobin from the filamentous fungi *Trichoderma reesei*, has been demonstrated to enhance the ER accumulation of fused proteins through the formation of PBs in transiently expressed *N. benthamiana* leaves [157]. The amphipathic property of HFBI also enables efficient protein purification by a surfactant-based aqueous two-phase system [157,158].

All three protein fusion partners (i.e., Zera^®^, ELP, and HFBI) promote large accumulations of ER-targeted recombinant proteins and induce the formation of ER-derived PBs, where these proteins are believed to be stably sequestered and protected. Plus, the unique physicochemical properties of ELP and HFBI make them valuable fusion partners that not only enhance the expression of recombinant proteins, but also assist in the fast and easy downstream purification [159,160]. Nevertheless, several recent studies reported discrepant effects between ELP and HFBI fusions. Although the C-terminal fusion of ELP to the influenza hemagglutinin H5 significantly increased the accumulation compared to the unfused H5 in transgenic tobacco plants, the HFBI fusion did not shown any positive effects on increasing the protein level [161]. When fused with AnPGI for transient expression in *N. benthamiana*, ELP enhanced the protein accumulation at both ER and vacuole, whereas HFBI impaired it at both locations. Additionally, impaired protein activity was initially reported by both fusions [132]. In the case of transient expression of the Dengue virus non-structural protein 1 (NS1) in *N. benthamiana*, fusion of ELP, but not HFBI, together with ER targeting greatly enhanced the recombinant protein accumulation, with an increase of up to 40-fold compared to the unfused version [118].

The engineered carrier molecule LicKM is derived from the thermostable lichenase (β-1,3-1,4-glucanase) of *Clostridium thermocellum*. This lichenase has a thermostable feature of maintaining activity at high temperatures, thus allowing for fast and cost-effective purification of its fused proteins by simple heat treatment [100]. LicKM was first adopted in the TMV replicon-based plant expression system, which subsequently has been used to generate high level transient expression of a number of LicKM-fused vaccine antigens in *N. benthamiana* [100,162,163]. Through a parallel comparison, fusion of four human growth factors (i.e., EPO, SCF, IL-3, and insulin-like growth factor-1 (IGF-1)) into the surface loop of LicKM resulted in a mild to dramatic increase in the expression and solubility in *N. benthamiana*. Additionally, these plant-made growth factors exhibited equivalent potencies to the commercial ones [101]. Nevertheless, it seems the effect of LicKM in the stable transgenic plants or with a non-viral binary vector expression system has not been evaluated so far.

Almost exclusively used in tobacco cell culture (e.g., BY-2), the fusion of target proteins to a hydroxyproline (Hyp)-*O*-glycosylated peptide (HypGP) tag has been demonstrated to dramatically boost the secretion and yield (up to 500-fold increase) of the recombinant proteins [85,86,93]. The design of HypGP tags is based on the Hyp-rich glycoproteins (e.g., arabinogalactan proteins (AGPs), extensins, and proline-rich proteins) that abundantly exist as cell wall structural glycoproteins unique to higher plants and green algae [164,165,166], and these tags are speculated to function as molecular carriers to facilitate the transport of the fused proteins across the plasma membrane and to protect against proteolytic degradation [87,93]. Different HypGP designs have been reported for the production of various biopharmaceutical proteins. The utilization of AGP-derived tandem repeat of (SP)_n_ (n usually ranges from 10 to 32) tags has led to high secretion yields of active recombinant therapeutic proteins in tobacco BY-2 cell culture, such as human interferon α2b (IFNα2) [85], human growth hormone (hGH) [86], and human stem cell factor (SCF) [42]. Another AGP-style tag (AP)_20_ has also been tested and showed similar potential to increase the secreted yield of the recombinant human α1-antitrypsin (AAT) in tobacco BY-2 cell culture [103]. In addition, the AGP-tag fusions have been shown to facilitate a prolonged half-life of the recombinant therapeutic proteins [85,86].

Although not designed specifically for increasing recombinant protein yields in plants, several carrier molecule fusions have been adopted and are routinely used in plant-based systems to achieve improved efficacy or an extended serum half-life of candidate vaccines, neutralizing antibodies, and other therapeutic proteins. For plant-based vaccine candidates, immunogenicity and the ability to elicit strong cellular and humoral immune responses are key considerations in the rational design of the recombinant antigens. The *Vibrio cholerae* cholera non-toxic B subunit (CTB) and the enterotoxigenic *E. coli* heat labile enterotoxin B subunit (LTB) are two well-characterized bacterial proteins with high immunogenicity but no toxicity, and they have been extensively used for the production of various chimeric antigens in non-plant systems [29]. Both CTB and LTB have also been adopted in plants and used as mucosal carriers for their translationally fused antigens to elicit immune responses. To date, a large number of plant-derived vaccines against human infectious diseases have been produced as chimeric antigens fused to CTB/LTB mainly for oral administration [19,28,167]. It has been well established in mammalian cell systems that fusion with the fragment crystallizable (Fc) domain of human immunoglobulin G (IgG), the human serum albumin (HSA), or the human transferrin, can markedly prolong the plasma half-life of the recombinant therapeutic proteins [168,169,170,171,172]. The Fc or transferrin fusion methods have been exploited in plant expression systems with promising results. For example, the expression of HIV p24 antigen fused with the Fc domain of human IgA in transgenic tobacco was reported, and this plant-made HIV p24-Fc recombinant antigen was able to form homodimers and induce cellular immune responses in mice [173]. Interestingly, the Fc fusion also dramatically increased the yield of HIV p24 compared to being expressed alone in tobacco [173]. The most widely used fusion part of Fc in plant expression systems appears to be that of the human IgG1. It has been used in the production of a number of bioactive vaccines, including a dengue fever vaccine candidate derived from the consensus domain III (cEDIII) of dengue glycoprotein E [174,175], and therapeutic proteins, such as human EPO [176,177,178,179], human osteopontin (OPN) [180], and human angiotensin-converting enzyme 2 (ACE2), against the severe acute respiratory syndrome coronavirus 2 (SARS-CoV-2) [181]. Fusion of human transferrin to the incretin hormone glucagon-like peptide-1 (GLP-1) has also led to robust accumulation of the protein and promising bioactivity in both transiently expressed and stable transgenic *Nicotiana* plants [114]. It seems the production of plant-based antibodies or therapeutic proteins based on HAS-fusion has not been reported yet.

### 2.5. Minimizing Proteolytic Degradation along the Secretory Pathway

Despite various developed strategies and platforms for the biopharmaceutical protein production with plant cells, proteolytic degradation remains a big hurdle that causes low yield or bioactivity. Plant cells contain a large number of endogenous proteases for normal cellular functions. The *N. benthamiana* genome was predicted to harbor over one thousand protease genes, only a small fraction of which have been functionally elucidated [182]. Both of these proteases are intracellularly and extracellularly localized, creating a challenging environment for maintaining the stability of the exogenous recombinant proteins [37,183,184]. As mentioned previously, cytosolic and lytic vacuoles are rich in proteases and, therefore, are often regarded as unsuitable sites for recombinant protein deposition. The naturally occurring protein storage organelles, such as PSVs or ER-derived PBs, are usually the ideal destinations for recombinant protein deposition in plants. Additionally, many pharmaceutical proteins require glycosylation for correct folding and desired bioactivity, making it inevitable for them to pass along the secretory pathway, where most post-transcriptional modification take place. Although targeting the recombinant protein to a specific organelle for accumulation or apoplast secretion has led to rapid and easy production of many recombinant proteins in plants, it is not a universal strategy that fits every scenario. Therefore, fully understanding the plant secretory pathway and its differences from that in mammalian cells will play an important role in improving recombinant protein production in plants.

#### 2.5.1. Native *N*-Glycosylation Sites and *N*-Glycan Occupancy

Eukaryotic cells are equipped with a variety of protein quality control mechanisms, among which the ER protein quality control (ERQC) mechanism is the best characterized. The ERQC ensures the proper folding of the secretory and transmembrane proteins before releasing them into the Golgi apparatus for further PTMs. By monitoring the folding status of a protein, ERQC retains and refolds the misfolded/incompletely folded proteins, and removes the terminally misfolded proteins through the ER-associated degradation (ERAD) pathway [109,185,186]. ERQC greatly relies on the *N*-glycosylation that initially takes place once the nascent polypeptide enters the ER lumen. The plant oligosaccharyltransferase (OST) complex catalyzes the *en bloc* transfer of the pre-assembled oligosaccharide, the three-branched Glc_3_Man_9_GlcNAc_2_, to the Asn residue in the consensus sequence or sequon Asn-X-Ser/Thr (X denotes any amino acid but Pro) of a protein. The attached *N*-glycan is recognized, processed, and bound by the lectin chaperones calnexin/calreticulin (CNX/CRT) that work in concert with additional chaperones and folding enzymes (e.g., binding immunoglobulin protein (BIP), protein disulfide-isomerase (PDI), and ER resident protein 57 (ERp57)), to aid in correct folding/refolding and inter/intramolecular disulfide bond formation [187,188,189]. Although the fundamentals of the ERQC mechanism are conserved among eukaryotes, differences do exist, such as the trimming of mannose residues on the *N*-glycan required for ERAD [109,186]. Despite the fact that the structural and functional characteristics are not fully understood, it is believed that the plant OST complex has unique preferences in the sequons, which lead to the underglycosylation of recombinant proteins in plants [190,191,192]. Although it has been speculated that underglycosylation will cause significant yield loss of the target proteins due to the important role *N*-glycans play during lectin chaperone-assisted folding, several recent studies do not corroborate this assertion. The rice endosperm accumulated HIV-neutralizing mAb 2G12 was found to be predominantly aglycosylated [193]. This substantial underglycosylation not only barely affected the yield and assembly of the antibody, but also led to a positive effect on its HIV-neutralizing activity. An OST derived from *Leishmania major* (LmSTT3D) was found to be able to effectively increase the *N*-glycan occupancy when co-expressed with recombinant glycoproteins and immunoglobulins. However, co-expression of LmSTT3D had little effect on target protein levels [194]. Instead, the presence of the native *N*-glycosylation sites, especially at the N-terminus, appears to be more important for the functional expression of glycoproteins in plants. For example, transient expression of an aglycosylated version of CTB as secretory protein resulted in very poor protein yield and massive tissue necrosis, while restoration of the single N-terminal *N*-glycosylation site led to an exceptionally high yield of CTB [195]. More recently, Shin et al. (2021) demonstrated that the substitution of the Asn residue at either one of the two *N*-glycosylation sites at the N-terminus of the truncated SARS-CoV-2 receptor binding domain (RBD) completely denied its expression as a secretory protein [196]. These results suggest that the intactness of the native *N*-glycosylation sites is more important than the *N*-glycan occupancy on these sites in terms of protein yield, although the exact molecular reasoning behind it remains to be elucidated. Nevertheless, the structures of *N*-glycans do play important roles in terms of the functionalities of most pharmaceutical glycoproteins.

#### 2.5.2. Chaperones and Folding Enzymes

As mentioned above, it is currently understood that differences in the *N*-glycan-involved ERQC exist between plants and mammals. In addition, the protein sequences of several major plant chaperones and folding enzymes, including CNX, CRT, BIP, PDI, and ERp57, have low similarity to their human homologs, suggesting a potential incompatible or insufficient plant endogenous folding machinery for exogenous proteins may exist [34]. Therefore, the folding of foreign recombinant glycoproteins in plant cells is considered to be more error prone than that of the native proteins, creating a potential bottleneck that may lead to the low protein yields. Co-expression of key chaperones derived from the same origins as the foreign proteins being expressed has been reported to improve folding and increase accumulation. For example, the accumulation of the enterohaemorrhagic *E. coli* type 3 secretion system (T3SS) effector protein Tir was significantly enhanced by co-expression of the CesT chaperone from the same bacterial secretion systemin both transiently transformed *N. benthamiana* and stable transplastomic tobacco plants [33]. Similarly, co-expression of the human lectin chaperone CRT markedly improved the protein yield of a soluble HIV-1 gp140 antigen while alleviating the ER stress response in *N. benthamiana* [34]. More recently, human CRT was found to specifically retain glycoproteins in ER-derived PB structures when co-expressing *N. benthamiana*, but this CRT-mediated ER retention exerts little effect on improving the folding of glycoprotein SARS-CoV-2 RBD [196]. In the same study, a truncated SARS-CoV-2 RBD with an eight-amino acid deletion on the C-terminus was also generated to avoid aberrant dimerization and aggregation potentially caused by an unpaired cysteine residue within the deleted region. This circumventing strategy led to a significant increase in the secretory protein level of RBD while also effectively eliminating dimerization [196]. Intriguingly, a single substitution of the same cysteine residue did not exhibit the same effect. Taken together, these results suggest that plant cells may lack the appropriate collection of chaperones and assisting catalysts to mediate the correct folding and disulfide bond formation of human or human-related pathogenic glycoproteins. It is therefore reasonable to speculate that the identification of and co-expression of specific native chaperones and folding enzymes from the same species origin as the targeted recombinant protein will likely have a profound impact on plant-based production of biopharmaceuticals.

#### 2.5.3. Deactivation of Proteases

Despite the large repertoire of proteases present in plants, not all of them are considered to be responsible for the proteolytic degradation of recombinant proteins because different types of proteases have tissue- and organelle-specific localizations, and the expression patterns of certain proteases are dependent upon the stage of growth and development, or stress conditions [182,183]. For example, the serine- and metallo-peptidases were found to be the major contributors to the extracellular degradation of human IgGs in the secretomes of tobacco cell cultures [197], whereas the ER-localized cysteine protease CysP6 was responsible for the degradation of recombinant human IL-10 in tobacco leaves [198]. Increased abundance of extracellular serine, cysteine, and aspartic proteases was reported in agroinfiltrated *N. benthamiana* leaves, suggesting that host immunity responses triggered degradation of the recombinant proteins [184]. Recently, two apoplastic abundant subtilisin-like serine proteases, NbSBT1 and NbSBT2, were shown to be responsible for the major proteolytic cleavage events happening to several recombinant human glycoproteins expressed in *N. benthamiana* [199]. Although functional identification of each protease remains a challenging task, these findings have greatly assisted in the development of strategies to keep the recombinant protein from undesired proteolysis along the plant secretory pathway. Directly blocking protease activity is believed to be the most effective strategy to protect target proteins. There are three main approaches to achieve this: (1) co-expression of protease inhibitors, such as SlCYS8, NbPot1, NbPR4, and HsTIMP; (2) protease gene knockdown/knockout using technologies such as RNAi, TALEN, or CRISPR/Cas9; and (3) pH regulation via the expression of the Matrix-2 proton channel protein. All three of these approaches have been well documented in the literatures [182,183,192].

In many cases, identifying the type(s) of proteases responsible for degrading a specific recombinant protein may be a difficult and time/labor-consuming task. In addition to the use of broad-spectrum protease inhibitors, one circumventing strategy is to modify the vulnerable region within the protein that is the target for cleavage to avoid proteolytic degradation. For example, despite the numerous examples of successful Fc-fused protein expression, proteolytic degradation at the hinge region of the Fc fusion has been a common issue in both plant-based platforms and other expression systems. A recent study demonstrated the enhanced stability of recombinant human EPO-Fc transiently produced in *N. benthamiana* by the substitution of four residues crucial to the Fc dimerization interface. The resulting monomeric EPO was stably expressed, maintained its bioactivity, and became resistant to proteolytic degradation [179]. Based on their findings, the authors of this study suggested that the weak point the Fc fusion technology is the exposure of the vulnerable region due to the lack of CL and highlighted the importance of the disulfide bond bridges between the CH1 and CL for the stability of native IgG structure [179]. Nevertheless, it is also possible that the exposure of the vulnerable hinge region is due to the lack of mucin-type *O*-glycosylation in plant cells, because the *O*-glycosylation at the hinge region of immunoglobulin heavy chains has been observed to protect against proteolytic digestion [200,201].

### 2.6. Silencing Suppressors and Silencing Knockout/Mutant Hosts

Plants have a sophisticated natural defense system to prevent pathogen invasion and remove foreign (pathogen) genetic materials, and this involves transcriptional gene silencing (TGS) and post-transcriptional gene silencing (PTGS) mechanisms [202,203,204,205,206]. In transgenic plants, expression of an introduced recombinant gene construct may be inhibited by TGS related methylation of the promoter sequence and, more frequently, the PTGS-caused degradation of its RNA transcripts, thereby greatly affecting the yield of recombinant proteins [35,207]. Several key effectors in the RNA silencing pathway have been identified, including the RNA-dependent RNA polymerases (RDRs), the Dicer-like (DCL) proteins, and the Argonaute (AGO) family proteins [205,206]. Disrupting the innate RNA silencing pathway may lead to an increased stability of the transgenic RNA and protein yield. Thus far, more than 70 silencing suppressor proteins have been identified from various plant viruses, most of which obstruct the formation of the siRNA-initiated effector complex, the RNA-induced silencing complex (RISC), through the interaction with either the siRNA, dsRNA, or the key effectors mentioned above [36,208,209]. Perhaps one of the most widely used viral silencing suppressor is P19 protein, first identified from *Tombusvirus* [210]. Numerous studies have reported boosted expression of recombinant proteins by co-expression of the P19 silencing suppressor [44,211,212,213,214,215,216].

Another effective strategy for increasing the level of recombinant protein production is through the full or partial repression of the RNA silencing pathway in the host plant. Butaye et al. (2004) first reported the use of Arabidopsis *RDR6* mutants (*sgs2* and *sgs3*) with silenced PTGS as a platform to achieve high-level transgene expression, and this genetic background was subsequently used to structurally characterize the OST subunits in Arabidopsis [191,217]. In addition to Arabidopsis, tobacco (*N. tabacum*) and its close relative *N. benthamiana*, have been employed, but they do not have a well-established mutant/mapping resource or precisely annotated genome database. Therefore, to use these plant species requires various genetic engineering processes to create PTGS deficiency. Improved production of recombinant proteins was reported in *N. benthamiana* by the RNAi-mediated simultaneous knockdown of the *DCL2* and *DCL4* genes [218]. However, the degree of suppression of both genes was largely dependent on the efficiency of the RNAi, which may account for the fact that only the simultaneous knockdown of *DCL2* and *DCL4* improved the production of recombinant proteins [218]. In subsequent studies, the *RDR6* gene function in *N. benthamiana* was knocked out completely (*ΔRDR6*) using the CRISPR/Cas9 method and a steady increase in recombinant protein accumulation was observed in the *ΔRDR6* background [219]. Despite originally being used for studies of plant development and antiviral mechanisms, several gene knockout plants generated by CRISPR/Cas9 have also been shown to be potential platforms for the heterologous expression of recombinant proteins. For example, the *N. benthamiana AGO2* knockout lines (*ago2*) created by CRISPR/Cas9 were hyper-susceptible to potato virus X (PVX), turnip mosaic virus (TuMV), and turnip crinkle virus (TCV), leading to the increased accumulation of virus-derived recombinant GFP [220]. In addition, mutant plants of *Glycine max* or *Medicago truncatula* with defective small RNA processing capabilities, such as the *Dicer-like3* (*Gmdcl1*), *Gmdcl4*, *Double-stranded RNA-binding2* (*GmDrb2*), and *Hua enhancer1* (*MtHen1*), have been successfully generated with Zinc finger nucleases (ZFNs), TAL-effector nuclease (TALEN), and CRISPR/Cas9 technologies [221,222]. Thus, these mutants may serve as promising host candidates for recombinant protein production, especially with the viral expression systems.

### 2.7. Deconstructed Viral Replicon-Based Vectors and Virus-like Particles (VLPs)

In addition to the traditional T-DNA based binary vector-mediated transient or stable transformation, expression of foreign proteins using plant viruses as vectors has also been extensively studied due to their ability to achieve a high copy number of target genes resulting from autonomous replication within host plant cells [223]. An early strategy for transgene delivery involved the direct inoculation of plants with either purified viral DNA or ssRNA prepared by in vitro transcription, which limited the rapid and large-scale implementation of this approach. The host specificity of each virus and the genetic instability of certain early viral vectors would often lead to low protein yield and rapid loss or silencing of the transgene fragment in the host cells. Finally, if the vector contains the full-length viral genome, there is the potential that this could allow assembly of fully functional recombinant viruses with the capacity to be infectious, disease causing, and a potential risk to the environment [224,225,226]. The biosafety concerns and the above-mentioned limitations of the full-virus strategy prompted the generation of the deconstructed viral replicon-based vector (referred to as deconstructed vector hereafter) system and the VLPs. These revolutionary inventions have turned plant pathogens (viruses and bacteria) into tremendously useful tools that are now extensively used for the easy and efficient production of recombinant proteins, such as therapeutic antibodies and viral vaccines, in addition to the rapid screening of promising targets from a collection of candidates.

The deconstructed vector essentially incorporates a viral replicon, composed of the genomic components only needed for episomal replication, into the T-DNA region of a binary vector to allow for efficient DNA delivery and infection initiation assisted by Agrobacterium or biolistic delivery. The gene of interest is usually introduced in place of the gene encoding the viral coat protein (CP) under the control of a subgenomic promoter. Therefore, upon infection of the host plant cells, the deconstructed viral genome can maintain its high copy number for high level protein production but cannot be encapsidated to form viral particles due to the lack of CP [10,14]. Among the pioneering designs of the deconstructed vectors are the two-component controllable or inducible expression system based on the genome of the geminivirus bean yellow dwarf virus (BeYDV) [227,228] and the suite of pro-vector modules, referred to as the magnICON system by Icon Genetics, built upon the genomes of two tobamoviruses TMV and turnip vein-clearing virus (TVCV) to give rise to functional RNA replicons when delivered into the host cells [88,229]. Around the same time, a deconstructed vector based on the potexvirus PVX was developed [230], and two separate groups also reported the successful construction of the TMV-based vectors, named TRBO and ‘launch vector’, respectively [100,231]. Since these initial reports, a number of significant improvements have been made allowing easier vector manipulation, expression of multiple target genes simultaneously, and importantly, higher recombinant protein yields [44,50,57,215,232,233,234,235,236]. Over the past decade, the use of improved deconstructed vectors has led to the rapid, high yield production of many valuable pharmaceutical proteins in plants [9,10,14]. In addition, they have been utilized as effective tools in the advanced genome-editing technologies that are based on the expression of various nucleases, such as ZFNs, TALENs, and CRISPR/Cas9 [52,237,238,239,240,241].

Plant-produced VLPs have emerged as promising alternatives for the production of many therapeutic proteins, notably candidate vaccines against infectious disease agents, such as influenza viruses, hepatitis B virus (HBV), HIV, HPV, and SARS-CoV-2 [12,16,242,243,244,245]. The formation of VLPs in plant cells is usually achieved by the expression of a specific structural protein from a selected animal or plant virus, such as influenza virus haemagglutinin (HA), HBV core antigen (HBcAg), the capsid proteins of the human HPV and norovirus viruses, and coat proteins of the plant PVX, TMV, or cowpea mosaic virus (CPMV). Expressed alone or fused with a peptide epitope, structural proteins can form VLPs via spontaneous self-assembly, resulting in a multivalent-antigen-presenting structure that mimics the morphological and immunological properties of the original virus but without any viral genetic material [246,247,248,249,250,251]. Therefore, plant-derived VLPs are considered safe and efficient in the immune response stimulation, and more importantly, exhibit great potential for the presentation of a wide range of candidate antigens through chimeric fusion. Successful production of the plant VLP-based vaccines or vaccine candidates in past decades has been well documented by previous reviews [4,7,12,16,29,252].

Most VLPs produced in plant cells are only tens of nanometers in diameter [253,254]. This is also true for the VLPs derived from plant capsid viruses, such as TMV, CPMV, and PVX [255,256]. In recent years, plant virus-derived VLPs have also been referred to as virus nanoparticles (VNPs), drawing more and more attention by nanotechnologists as the next-generation diagnostic and therapeutic bioreagents for imaging and drug delivery [13,14,15,257].

Since the onset of the worldwide coronavirus disease 2019 (COVID-19) pandemic caused by SARS-CoV-2, the development of rapid, safe, and cost-effective SARS-CoV-2 therapeutics, such as antiviral antibodies and vaccines, has become an urgent global demand. Previous studies have shown that the ACE2 receptor protein, which mediates viral entry into human cells, can be engineered as a promising therapeutic agent for attenuating viral infection and replication [258,259,260,261]. One of the recombinant human ACE2 proteins has already passed Phase 2 clinical trials (NCT04335136). Conversely, developing subunit or VLP vaccines based on critical SARS-CoV-2 antigens, such as the S1 subunit of the spike (S) glycoprotein (or smaller RBDs within it) that facilitates virus entry by specific binding to the ACE2 on cell surface, has been considered as a faster and safer alternative than the traditional approach of vaccine development [244,262,263]. The use of plant-based platforms for the production of antibodies and vaccine candidates against SARS-CoV-2 has also been implemented [16,244]. The promising results of plant-produced ACE2-Fc fusion protein as a potential therapeutic candidate against SARS-CoV-2 was recently reported [181]. Some plant-based vaccine candidates have also been developed and have already made their way into Phase 2/3 clinical trials. For example, a tobacco-produced vaccine candidate based on the SARS-CoV-2 protein S1 subunit was developed by Kentucky BioProcessing, a subsidiary of British American Tobacco [244] and is currently under Phase 2 clinical trials (NCT04473690). Notably, a self-assembled VLP presenting the full-length S glycoprotein was produced in *N. benthamiana* and exhibited safe immunogenicity and efficacy against SARS-CoV-2 in preclinical and early phase clinical trials [245,264]. With the recent completion of Phase 3 trials (NCT04636697), this COVID-19 VLP vaccine developed by Medicago may become the first plant-based vaccine to be approved for use in humans.

## 3. Strategies to Improve Protein Activity/Quality

Glycosylation is the most important post-translational modification that ensures the integrity and functionality of the glycoproteins among eukaryotes. In plants and mammals, *N*-glycosylation and *O*-glycosylation are the two most important types of protein glycosylation, and both are subject to extensive processing steps along the secretory pathway leading to the addition of various structurally diverse glycans onto proteins. For many human glycoproteins, such as antibodies and hormones, these decorating glycans are essential to their physicochemical properties, including efficacy, solubility, stability, and proteolytic resistance [265,266]. Therefore, providing appropriate glycan additions to recombinant glycoproteins is indispensable for the high-quality production of biopharmaceuticals in plants.

**Figure 1:**
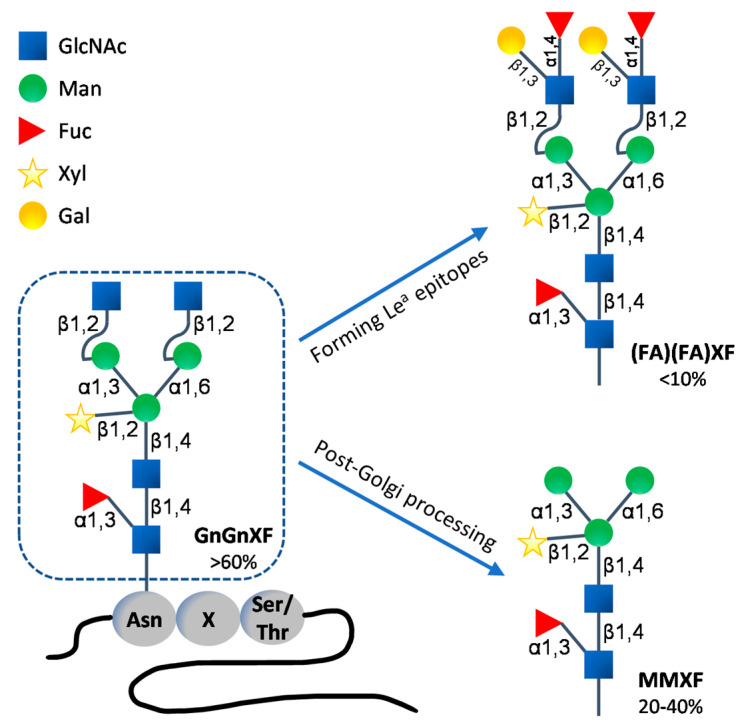
Typical plant-derived *N*-glycan structures. In plant cells, the GnGnXF structure accounts for the majority of the *N*-glycoforms. The second most abundant structure is the paucimannosidic MMXF with truncated terminal GlcNAc residues generated by specific β-*N*-acetylhexosaminidases located at either the vacuole or the plasma membrane/apoplast. Although relatively low in abundance, the (FA)(FA)XF structure with terminal Lewis A (Le^a^) epitopes generated in the *trans* Golgi compartment is commonly found in plants. All three *N*-glycoforms presumably carry plant specific α1,3-linked fucose and β1,2-linked xylose residues. GlcNAc, *N*-acetylglucosamine; Man, Mannose; Fuc, Fucose; Xyl, Xylose; Gal, Galactose.

**Figure 2:**
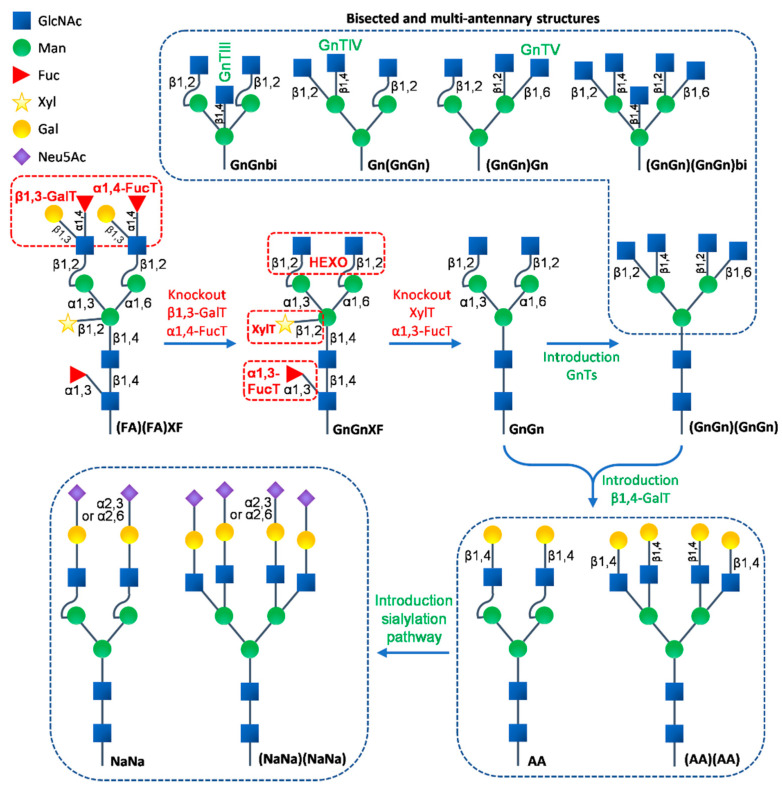
Glycoengineering of plants for the production of human-type complex *N*-glycan structures. To eliminate plant-specific α1,3-linked fucose and β1,2-linked xylose residues, two enzymes, β1,2-xylosyltransferase (XylT) and core α1,3-fucosyltransferase (α1,3-FucT), have been knocked down/out, generating the common eukaryotic core GnGn. Furthermore, a more homogenous GnGn can be achieved by the elimination of β1,3-galactosyltransferase (β1,3-GalT) and α1,4-FucT, the enzymes responsible for the generation of Le^a^ epitopes, in addition to the knockout of two β-*N*-acetylhexosaminidases (HEXO), which catalyze the trimming of the terminal GlcNAc residues, to prevent the formation of the paucimannosidic structures. Various bisected and multi-antennary structures can be generated by introducing the *N*-acetylglucosaminyltransferases (GnTs) that are absent in plants (i.e., GnTIII-GnTV). The elaborate introduction of β1,4-GalT and enzymes involved in the human sialylation pathway results in β1,4-galactosylation followed by terminal sialylation (via α2,3- or α2,6-linkage) to GnGn or multi-antennary structures, such as (GnGn)(GnGn), generating fully sialylated human-type *N*-glycans. Neu5Ac, *N*-acetylneuraminic acid.

The differences in the *N*-glycan structures on proteins between plants and animals have been known for a long time and these differences have been among the major safety and quality concerns in the use of plant-produced recombinant pharmaceutical proteins for humans and animals. From the ER to the *cis* Golgi apparatus, the *N*-glycan processing steps are highly conserved between plants and animals. Significant differences in further *N*-glycan maturation start emerging in the *medial* Golgi and along the secretory pathway, giving rise to distinct complex *N*-glycans due to the differences in the genome-encoded glycosyltransferases between plants and animals [17,108,166,190]. Plant-derived *N*-glycans typically carry β1,2-xylose and core α1,3-fucose residues to form the GnGnXF (GlcNAc_2_XylFucMan_3_GlcNAc_2_) structures, which sometimes become further extended with Lewis-A (Le^a^ or FA) epitopes to form (FA)(FA)XF (i.e., the addition of β1,3-galatose and α1,4-fucose residues on terminal GlcNAc residues, Figure 1). In addition, post-Golgi processing of the GnGnXF *N*-glycans generates paucimannosidic MMXF (Man_3_XylFucGlcNAc_2_) structures by removing the terminal GlcNAc residues (Figure 1). Although structurally less diverse compared to animals, these plant specific complex *N*-glycan epitopes have raised concerns as a potential source of undesired immunogenicity in animals. Moreover, plants lack typical mammalian complex *N*-glycan modifications, such as the formation of bisected and branched multi-antennary structures and the further elongation via galactosylation and sialylation (Figure 2), which are now known to be essential to the potency and serum half-life of therapeutic glycoproteins [7,267]. During the past several decades, numerous efforts have been made towards identifying and eliminating limitations associated with glycosylation in plant molecular farming, with the goal of producing recombinant proteins with optimal characteristics through tailored glycosylation [17,21,190,192].

### 3.1. Elimination of Plant Specific N-Glycan Structures

The addition of β1,2-xylose and core α1,3-fucose is catalyzed by β1,2-xylosyltransferase (XylT) and core α1,3-fucosyltransferase (α1,3-FucT), respectively. Both enzymes are unique in plants. Blocking both enzymes to generate GnGn (GlcNAc_2_Man_3_GlcNAc_2_), the core *N*-glycan structure shared between plants and mammals, is the first step towards the engineering of mammalian type *N*-glycans in plants (Figure 2). Arabidopsis T-DNA insertion triple mutant lines have been generated and have shown complete deficiency of both XylT and FucT activities [268]. Both enzymatic activities were subsequently eliminated with RNAi technology in other plant species that are considered more suitable for molecular farming, such as *N. benthamiana* [269], rice [270], and *Lemna minor* [271]. Among these glycoengineered plant hosts, the *N. benthamiana* line ΔXT/FT has been used for the expression of numerous recombinant proteins, including recombinant human immunoglobulins, enzymes, and viral antigens. No detectable xylose or α1,3-fucose residues were found and GnGn was determined to be the predominant *N*-glycan structure on these proteins. More importantly, some of the recombinants showed equivalent or even enhanced potency compared to their counterparts produced in mammalian cells [196,272,273,274,275,276]. Additionally, the ΔXT/FT *N. benthamiana* has served as a ‘jumping off’ background for additional glycoengineering designs. With the advancement of CRISPR/Cas9 technology, the new generation of the XylT and FucT deficient lines of *N. benthamiana* [277], *N. tabacum* BY-2 cells [278,279], and rice [280] were created by simultaneous knockout of multiple glycosyltransferases genes at the genome level. These glycoengineered plants provide diverse platforms for the production of pharmaceutical glycoproteins devoid of plant-type residues or containing human-type *N*-glycosylation.

Although not as abundant as the GnGnXF structure, the paucimannosidic MMXF generated at the late stages of the *N*-glycosylation pathway is sometimes considered as an undesired *N*-glycan epitope that negatively affects the bioactivity or potency of the recombinant proteins for therapeutic applications [281]. In both Arabidopsis and *N. benthamiana*, MMXF is formed mainly in the vacuoles and apoplasts through the trimming of terminal GlcNAc residues by at least two β-*N*-acetylhexosaminidases (HEXO), the vacuole-located HEXO1 and plasma-membrane-located HEXO3 [281,282]. Previous results have shown that the Arabidopsis *HEXO* mutant and *N. benthamiana HEXO* RNAi knockout line effectively prevent the formation of the paucimannosidic MMXF *N*-glycan [281,282]. However, the paucimannosidic structure MM (Man_3_GlcNAc_2_ or M3) has been reported to be indispensable and superior in mannose receptor (MR)-mediated cellular delivery of biopharmaceutics because of its high MR-binding efficiency and relatively low clearance rate [89]. Carrot suspension cell-produced Elelyso (i.e., recombinant human β-glucocerebrosidase) is an example of a recombinant protein with the paucimannosidic type *N*-glycan [131]. The human acid-alpha glucosidase (GAA) is also a therapeutic protein used for enzyme replacement therapy of Pompe disease. Using the glycoengineered Arabidopsis cell culture derived from a mutant plant lacking functional α1,3-mannosyltransferase (ALG3), the enzyme involved in the early assembly of the oligosaccharide precursor, the recombinant human GAA was produced with MM comprising the majority of the *N*-glycan structures [89]. Therefore, for therapeutic proteins designed for cellular delivery through MR, such as those used for enzyme replacement therapies, the paucimannosidic *N*-glycan modification is considered to be beneficial. Finally, the elimination of the Le^a^ epitope was successfully implemented by the targeted knockout of the two genes encoding α1,4-FucT and β1,3-galactosyltransferase (β1,3-GalT) in moss [283] (Figure 2).

### 3.2. Introduction of Humanized N-Glycosylation Pathway in Plants

The lack of human-type *N*-glycan structures in plants is due to the absence of a collection of enzymes that are responsible for several processes: (1) the addition of core α1,6-fucose residue; (2) the formation of multi-antennary structures; (3) β1,4-galactosylation on the GlcNAc residues; and (4) the subsequent α2,3- or α2,6-sialylation on the galactose residues. Although the majority of human glycoproteins carry a core α1,6-fucose residue on their complex *N*-glycans, recent studies have found that the absence of the core fucose residue significantly enhanced the effects of many antibodies, and that both core α1,3- and α1,6-fucose similarly affected the antibody binding to the Fcγ receptors [108,284,285]. It has also been reported that both types of fucosylation also negatively affect the abundance of galactosylated *N*-glycans on a mAb cetuximab (Cx-IgG) [286]. There are few comparative studies on the effects of core α1,6-fucose on other types of human therapeutic glycoproteins. Nevertheless, generation of afucosylated recombinant antibodies is considered as an effective strategy to improve therapeutic potentials [285]. To date, very limited attention has been focused on the introduction of core α1,6-fucose into the plant expression systems.

The common human *N*-glycan multi-antennary structures, including the bisected (GnGnbi), tri-antennary [(GnGn)Gn or Gn(GnGn)], and tetra-antennary [(GnGn)(GnGn)], are formed by the addition of diversely linked GlcNAc residues on the core mannose residues through the action of a series of *N*-acetylglucosaminyltransferases (GnTs), i.e., GnTI-GnTV (Figure 2). Among the five characterized GnTs, plants only possess active GnTI and GnTII, which account for the transfer of the two β1,2-GlcNAc residues. GnTIII-GnTV activities do not occur in plants. The GnTIII is responsible for the addition of β1,4-GlcNAc residue to the β1,4-mannose residue to form GnGnbi, whereas GnTIV and GnTV respectively add β1,4-GlcNAc to α1,3-mannose and β1,6-GlcNAc to α1,6-mannose, forming Gn(GnGn), (GnGn)Gn, and (GnGn)(GnGn) structures [108] (Figure 2). The *in planta* biosynthesis of glycoproteins with both bisected and branched complex *N*-glycans was achieved by the sequential expression of the human GnTIII, GnTIV, and GnTV enzymes fused to different sub-Golgi targeting signals [176]. The data from this and earlier studies also confirmed that the addition of the bisecting GlcNAc can block further processing of the *N*-glycans in both plants and mammals, and suggested the importance of GnTIII activity to be introduced at a late stage of *N*-glycan processing [108].

β1,4-galactosylation is a crucial intermediate step to glycoprotein sialyation because it provides the β1,4-linked galactose residues that serve as the acceptor substrate for the sialic acid residue *N*-acetylneuraminic acid (Neu5Ac). Glycoengineering on the *N. benthamiana* ΔXT/FT background for humanized β1,4-galactosylation was accomplished by the stable expression of the human β1,4-GalT targeted to the *trans* Golgi compartment (^ST^GalT). The results showed fully galactosylated *N*-glycan structures (AA, Figure 2) on transiently expressed human mAbs and enzymes [287]. Despite these promising results, the stably generated ^ST^GalT plants had several drawbacks, including large amounts of incompletely processed *N*-glycans, changes in the reactivity of cell wall carbohydrate epitopes, and stress-related phenotypic alterations. These findings suggested that there was interference with endogenous glycosyltransferases and/or other unfavorable consequences caused by the overexpression of exogenous glycosyltransferases [288]. The optimization of the ^ST^GalT expression strategy was subsequently performed by testing eight different promoter/terminator combinations in *N. benthamiana*, and the results showed that the efficiency of homogenous di-galactosylation of recombinant human mAbs could be improved by moderate expression of ^ST^GalT driven by less strong promoters [289]. Moreover, another prominent issue with the ^ST^GalT plant is the insufficient galactosylation (17% of *N*-glycans) on secreted proteins [288]. Recently, an apoplast localized β-galactosidase from *N. benthamiana*, NtBGAL1, was characterized and demonstrated to be actively involved in the trimming of not only the endogenous β1,3-galactose residues on the Le^a^ epitopes, but also the β1,3- and β1,4-galactose residues, respectively, on mucin-type *O*-glycans and *N*-glycans that do not naturally exist in plants. Suppression of NbBGAL1 activity significantly improved the yield of β1,4-galactosylated *N*-glycans by human GalT for several recombinant glycoproteins [286].

In humans, protein sialyation is performed by α2,6-sialyltransferase (α2,6-ST/ST6) or α2,3-ST/ST3, which adds the active form of Neu5Ac, cytidine monophospho (CMP)-Neu5Ac, to the terminal β1,4-galactose residues through α2,6- or α2,3-linkage [108,290]. Compared to the earlier plant glycoengineering, such as the elimination of plant specific xylose and fucose residues and the introduction of β1,4-galactosylation, the introduction of *N*-glycan sialylation in plants is much more challenging because of multiple constraints, such as the lack of the CMP-Neu5Ac biosynthesis and its related transporter and transferase [267]. Castilho et al. [291] first introduced a functional CMP-Neu5Ac biosynthesis pathway in Arabidopsis. Efficient *N*-glycan sialylation on recombinant glycoproteins was accomplished by the organized co-expression of six mammalian enzymes involved in various stages of the sialylation pathway in *N. benthamiana* [267,274]. As an escalated attempt at engineering, the coordinated co-expression of eleven mammalian proteins responsible for the branching, galactosylation and sialyation of the *N*-glycosylation was attempted in a *N. benthamiana* ΔXT/FT background, and led to the successful production of the recombinant human EPO decorated with bi-, tri-, and tetra-sialylated complex *N*-glycans and without plant specific residues [178] (Figure 2). The sialylated *N*-glycan structures were also successfully generated on secreted recombinant proteins accumulated in the apoplast of *N. benthamiana*. Intriguingly, the terminal sialylation was found to effectively protect the β1,2-GlcNAc residues from HEXO activities, thus preventing the formation of the paucimannosidic *N*-glycan structure on the secreted proteins [292]. To fully simulate the human sialylation, diversely linked (α2,6 and α2,3) sialylation together with polysialylation was achieved through the combinational strategy of stable and transient expression of a suite of enzymes for sialylation and two human polysialyltransferases (polySTs) for polysialylation in ∆XT/FT *N. benthamiana* [293]. These great milestone endeavors have demonstrated conclusively the feasibility of *in planta* production of the most complex human-type *N*-glycans, while also suggesting the high tolerance of plants to the manipulation in the *N*-glycosylation pathway, putting plants one step closer to the frontlines of the industrial production of human pharmaceutical proteins with diversified *N*-glycans.

### 3.3. Introduction of Human Mucin-Type O-Glycosylation in Plants

The mucin-type *O*-glycosylation is the most abundant *O*-glycosylation commonly found on human secretory proteins (e.g., mucin). It is initiated by the attachment of *N*-acetylgalactosamine (GalNAc) residues to Ser or Thr residues catalyzed by a family of enzymes named UDP-GalNAc polypeptide *N*-acetylgalactosaminyltransferases (GalNAc-Ts). The GalNAc residues are further elongated and even branched with other sugar residues, such as GlcNAc, galactose, and Neu5Ac, by various glycosyltransferases to generate highly diverse *O*-glycans [265]. Unlike *N*-glycosylation, which is partially conserved between plants and animals, plant *O*-glycosylation is fundamentally distinct from the human mucin-type *O*-glycosylation due to the lack of the whole catalytic machinery [266]. Instead, plant *O*-glycosylation is characterized by the attachment of arabinogalactan polysaccharides or arabino-oligosaccharides to the Hyp residues on the Hyp-rich glycoproteins, mainly the AGPs and extensins. To a lesser extent, attachment of a single galactose residue to the Ser residues occurs for specific proteins [166,190,265]. Therefore, the significant structural difference in *O*-glycans prompted the need of ‘humanizing’ the *O*-glycosylation for plant molecular farming.

Several pilot studies have reported the successful introduction of multiple enzymes responsible for the biosynthesis, transport, and attachment of UDP-GalNAc of the mucin-type *O*-glycosylation pathway into plants. These glycoengineered plants were able to produce the core 1 structure of mucin-type *O*-glycans without sialylation [294,295]. Eventually, the co-expression of more than ten genes involved in the mucin-type *O*-glycosylation and sialylation pathways in *N. benthamiana* allowed two recombinant human proteins (i.e., EPO-Fc and IgA1) to be successfully generated with both sialylated mucin-type core 1 *O*-glycans and sialylated *N*-glycans [177,273]. This is yet another major milestone of plant glycoengineering and underscores again the great potential of plants as production factories of recombinant glycoproteins with simultaneously added humanized *N*- and *O*-glycosylations.

## 4. Future Perspectives

With promising accomplishments in various aspects of pharmaceutical protein production in plants, including engineered plant lines and cells cultures, developed expression systems, enhanced yield and solubility (quantity), and improved glycosylation (quality), plant molecular farming has moved from an innovative concept to a realized practical platform with agricultural and pharmaceutical applications. However, due to the nature of plant cells, there are still barriers that keep plant expression platforms from being fully adopted as alternatives to mammalian cells for the production of most pharmaceuticals. When expressed in plant cells, the first challenge the nascent recombinant protein faces after entering ER is to maintain its native structure through correct folding and disulfide bridge formation because the aberrantly folded protein either forms nonfunctional aggregates or is degraded via ERAD [109,186]. Recent studies have suggested the incompatibility of plant endogenous chaperone machinery for the correct folding and disulfide bond formation of heterologous glycoproteins, which led to great ER stress and low protein yield [34,196]. The exact molecular mechanism of the native chaperon assisted folding of each recombinant protein is also largely unknown. Therefore, a great amount of work is needed to improve the folding capabilities of plant folding machinery for increased yield of exogenous proteins and the prevention of aberrant oligomer deposition.

Although the revolutionizing achievements in glycoengineering have made humanized *N*- and *O*-glycan production in plants a reality [17,190], most remain proof-of-concept demonstrations and the *N. benthamiana* ΔXT/FT background still appears to be the most used host to generate antibodies with simple GnGn *N*-glycan modification, even in more recent studies [296,297]. This may indicate that there are still imperfections in the system that impede the universal adoption of the expression platforms that generate the most sophisticated complex glycan profiles, such as the sialylated *N*-glycosylation and mucin-type *O*-glycosylation. The coordinated, fine-tuned, and correctly localized expression of each enzyme involved in the glycosylation pathway has been demonstrated to be extremely important for the successful glycan production and prevention from enzyme interference when introduced in plants [176,178,267,289]. In terms of *O*-glycosylation, the GalNAc-T family contains at least twenty members in humans, and fully deciphering the acceptor substrate specificity for each isoenzyme is still on the way, suggesting the possible inefficient initiations of mucin-type *O*-glycosylation when inappropriate or insufficient GalNAc-Ts are used in plants [273,298]. In addition, the issue of low sialylation efficiency and high heterogeneity of the plant-generated mucin-type *O*-glycans and the concern about the presence of plant-endogenous Hyp-linked *O*-glycans on the recombinant proteins produced in glycoengineered plants need to be further addressed. Thus, it will still be a challenging but promising task to improve and develop more glycoengineered plant lines and cell cultures that provide customized mammalian glycosylation with high efficiency and homogeneity, and free of adverse effects on the host cells.

When compared to currently established cell expression platforms (e.g., mammalian, bacterial, insect, and yeast) used as the industrial standards, various plant production platforms still face multiple challenges, such as relatively low yield and high cost of downstream processing, that have greatly slowed their translation into commercial applications. These challenges have been well discussed from either the industrial production or clinical development point of view [22,82,299,300,301]. In addition, the regulation and biocontainment of the transgenic plants used for molecular farming, especially in large-scale production, should also be seriously dealt with to eliminate cross-transgene contamination and unwanted gene flows from transgenic plants to the ecosystem [144]. Nonetheless, as these challenges continue to be overcome, it is believed that more and more plant-produced pharmaceuticals will make the leap from laboratory benches to industrial production lines.

## Figures and Tables

**Table 1:** Examples of the seed endosperm accumulation of recombinant pharmaceutical proteins in stable transgenic cereal plants.

Recombinant Protein ^1^	Origin	Host Plant	Yield	References
Derivatives of Japanese cedar pollen allergens Cry j 1 and Cry j 2	*Cryptomeria japanica*	*O. sativa*	Up to 60 µg/seed	[61,62,63]
Derivatives of house dust mite allergens	*Dermatophagoides* spp.	*O. sativa*	Up to 90 µg/seed	[64,65,66]
Birch pollen allergens	Birch	*O. sativa*	Up to 550 µg/seed	[67,68]
LTB–COE	PEDV	*O. sativa*	1.3% of endosperm TSP	[69]
CTB	*Vibrio cholerae*	*O. sativa*	Up to 30 µg/seed	[70]
CTB-vaccine antigens (As14 and As16)	*Ascaris suum*	*O. sativa*	50 µg/g seed, 1.5 µg/seed	[71,72]
Chimeric HBV surface antigen SS1	HBV	*O. sativa*	31.5 ng/g seed	[73]
AMP Cecropin A	Insects	*O. sativa*	Up to 6 μg/g seed	[74]
IL-10	Human	*O. sativa*	1.2 mg/g seed, 219 μg/seed	[75,76]
Anti-HIV mAb 2G12	Human	*Z. mays*	Up to 60 µg/g seed	[58]
Anti-HIV mAb 2G12	Human	*H. vulgare*	160 μg/g seed	[59]
Cathelicidin AMP LL-37	Human	*H. vulgare*	Up to 550 μg/kg seed	[60,77]

^1^ Abbreviations: LTB-COE, a fusion protein composed of the B-subunit of the *E. coli* heat-labile enterotoxin (LTB) and a synthetic core-neutralizing epitope (COE) of porcine epidemic diarrhea virus (PEDV); CTB, cholera toxin B subunit; HBV, Hepatitis B virus; AMP, antimicrobial peptide; IL, interleukin; HIV, human immunodeficiency virus; mAb, monoclonal antibody.

**Table 2:** Selected examples of signal peptide sequences used for the expression of recombinant pharmaceutical proteins in plants.

Signal Peptide			Heterologous Expression		References
Name ^1^	Amino Acid Sequence	Origin	Recombinant Protein ^2^	Host	
Ramy3sp	MKNTSSLCLLLLVVLCSLTCNSGQA	*O. sativa*	hG-CSF, hzAb, hGH, mGM-CSF, CCP-mAb	Rice cell culture	[94,95,96,97,98,99]
33KDsp	MAALSQLVLVTAFLAAALLPLGMAA	*O. sativa*	mGM-CSF	Rice cell culture	[83]
SS^Pr1^	MLPSFLLVSTLLLFLVISHSCRA	*N. benthamiana*	HA and NA of H5N1, EPO, SCF, IL-3, IGF-1, IFNγ	*N. Benthamiana*	[84,100,101,102]
NbSS^Ext^	MGKMASLFATLLVVLVSLSLASESSA	*N. benthamiana*	IFNγ	*N. Benthamiana* and its cell culture	[84]
NtSS^Ext^	MGKMASLFASLLVVLVSLSLASESSA	*N. tabacum*	IFNα2, AAT, hGH, SCF	tobacco BY-2 cell culture, tobacco hairy root culture	[42,85,86,103]
SS^VspA^	MKMKVLVFFVATILVAWQCHA	*G. max*	SEAP, Ebola GP1, IFNγ	*N. Benthamiana*, tobacco TN1 cultured cells	[84,104,105]
ZmCKX1sp	MAVVYYLLLAGLIACSHA	*Z. mays*	hLL-37	*N. Benthamiana*, Barley	[60]
LeB4sp	MSKPFLSLLSLSLLLFTSTCLA	*V. faba*	2G12, HA of H5N1	*N. Benthamiana*, Barley	[59,106]
PDIsp	MAKNVAIFGLLFSLLVLVPSQIFA	*M. sativa*	AACT, IFNγ	tobacco BY-2 cell culture	[84,107]

^1^ Name annotation: Ramy3sp, signal peptide of rice α-amylase 3D; 33KDsp, signal peptide of a 33KD secretory protein encoded by *Os04g0659300*; SS^Pr1^, signal peptide of *N. Benthamiana* pathogenesis related protein 1; SS^Ext^, signal peptide of extensin; SS^VspA^, signal peptide of soybean vegetative storage protein; ZmCKX1sp, signal peptide of maize cytokinin dehydrogenase 1; LeB4sp, signal peptide of *Vicia faba* legumin B4; PDIsp, signal peptide of *Medicago sativa* protein disulfide isomerase. ^2^ Recombinant protein abbreviations: hG-CSF, human granulocyte-colony stimulating factor; hzAb, anti-TAG 72 humanized antibody fragments; hGH, human growth hormone; mGM-CSF, mouse granulocyte-macrophage colony-stimulating factor; CCP-mAb, cyclic citrullinated peptide-specific monoclonal antibody; HA, hemagglutinin; NA, neuraminidase; H5N1, avian influenza virus subtype H5N1; EPO, erythropoietin; SCF, human stem cell factor; IL-3, interleukin 3; IGF-1, insulin-like growth factor-1; IFN, human interferon; AAT, human protease inhibitor α1-antitrypsin; SEAP, human secreted alkaline phosphatase; hLL-37, small human AMP LL-37; AACT, human α1-antichymotrypsin.

**Table 3:** Examples of transient expression of ER-accumulated recombinant pharmaceutical proteins in *N. benthamiana*.

Recombinant Protein	Origin	Expression System ^1^	Yield	References
Envelope protein subunits	Dengue virus	Viral replicon-based	Up to 600 mg/kg FW	[116,117]
Non-structural protein 1	Dengue virus	Binary vector-based	445 mg/kg FW	[118]
Glycoprotein subunit 1	Ebola virus	BeYDV replicon-based	50 mg/kg FW	[105]
HA	H5N1	TMV replicon-based	60 mg/kg FW	[102]
HA	H5N1	Binary vector-based	Up to 0.02% TSP	[106]
HA-Neuraminidase	Newcastle disease virus	Binary vector-based	Up to 3000 mg/kg FW	[119]
Envelope protein	Yellow fever virus	TMV replicon-based	NA	[120]
HIV envelope protein subunit, anti-HIV mAbs and scAb	HIV, human	Binary vector-based	80–600 mg/kg FW	[121]
anti-HIV mAb 2G12	Human	CPMV based replicating and non-replicating	Up to 105.1 mg/kg FW	[122]
EPO	Human	Binary vector-based	500 μg/g TSP	[43]
Growth factors	Human	TMV replicon-based	10–250 mg/kg FW	[101]
Butyrylcholinesterase	Human	TMV replicon-based	NA	[123]
IFNγ	Human	BaMV replicon-based	119 mg/kg FW	[44]
Colorectal cancer antigen GA733-2-Fc	Human	BCTV replicon-based	NA	[124]
AMPs	Bacterium, Fungus, Animals	Binary vector-based	20–565 mg/kg FW	[125]

^1^ Abbreviations: BeYDV, bean yellow dwarf virus; TMV, tobacco mosaic virus; CPMV, cowpea mosaic virus; BaMV, bamboo mosaic virus; BCTV, beet curly top virus.

## Data Availability

Not applicable.
